# Impact of Admission White Blood Cell Count on Short- and Long-term Mortality in Patients With Type A Acute Aortic Dissection: An Observational Study

**DOI:** 10.1097/MD.0000000000001761

**Published:** 2015-10-23

**Authors:** Xiaohan Fan, Bi Huang, Haisong Lu, Zhenhua Zhao, Zhinan Lu, Yanmin Yang, Shu Zhang, Rutai Hui

**Affiliations:** From the State Key Laboratory of Cardiovascular Disease, Department of Cardiology (XF, BH, ZL, YY, SZ, RH); and Department of Cardiovascular Surgery, Fuwai Hospital, National Center for Cardiovascular Diseases, Peking Union Medical College, Beijing, China (HL, ZZ).

## Abstract

Studies have shown inflammation is involved in the development of acute aortic dissection (AAD). The hypothesis that white blood cell count (WBCc) on admission may have an impact on the short- and long-term outcomes of type A AAD was tested in a large-scale, prospective observational cohort study.

From 2008 to 2010, a total of 570 consecutive patients with type A AAD in Fuwai hospital were enrolled and were followed up. Baseline characteristics and WBCc on admission were collected. The primary outcomes were 30-day and long-term all-cause mortality.

During a median of 1.89 years of follow-up, the 30-day and long-term all-cause mortality were 10.7% and 6.5%, respectively. Univariate Cox regression analysis identified admission WBCc as an independent predictor of 30-day mortality when considered as a continuous variable or as a categorical variable using the cutoff of 11.0  × 10^9^ cells/L (all *P* < 0.05). After adjustment for age, sex, C-reactive protein, d-dimer, and surgical intervention, elevated admission WBCc (>11.0 × 10^9^ cells/L) remained an independent predictor of 30-day mortality of AAD (hazard ratio = 3.31, 95% confidence interval 1.38–7.93, *P* = 0.007). No impact of admission WBCc was observed on the long-term all-cause mortality.

In conclusion, elevated admission WBCc may be valuable as a predictor of 30-day mortality, and may be useful in the risk stratification of type A AAD during hospitalization.

## INTRODUCTION

Type A acute aortic dissection (AAD) is a devastating cardiovascular condition with high mortality and identification of risk factors for prognosis is of great value for risk stratification in patients with type A AAD. In recent decade, studies have shown that inflammation was not only involved in the pathogenesis of AAD,^1–5^ but also played a part in the outcomes in patients with AAD. Some inflammatory biomarkers, such as C-reactive protein (CRP),^6,7^d-dimer,^6,8,9^ and fibrinogen^10^ have been shown to be related with the prognosis in patients with AAD. White blood cell count (WBCc) is a commonly used nonspecific marker of acute inflammatory response and limited studies with relatively small sample size evaluated the impact of WBCc on the in-hospital mortality in patients with AAD but reached inconsistent conclusion.^8,11^ Moreover, there is lack of data regarding the association of WBCc with the long-term outcomes in patients with AAD. Therefore, we conducted a prospective observational cohort study with relatively large sample size to evaluate the impact of admission WBCc on the short- and long-term outcomes in patients with type A AAD.

## METHODS

A series of consecutive patients who presented to the cardiac emergency center for suspicious AAD were screened from January 2008 to December 2010 in FuWai Hospital (National Center for Cardiovascular Diseases in China). Diagnosis of AAD was confirmed by aorta angiography using multidetector computed tomographic (CT) scanning. The AAD was defined as acute if chest pain or other related symptoms were present <2 weeks before presentation to our hospital. The Stanford type A (DeBakey I and II) dissection was defined as involving the ascending aorta and/or aortic arch according to previously published criteria.^12^ Patients were excluded if they had clear etiology, such as Marfan syndrome, Loeys-Dietz Syndrome, or iatrogenic AAD secondary to cardiac surgery or thoracic endovascular aortic repair, or a history of operation for AAD or chronic dissections. This study was approved by the ethical committees of Fuwai Hospital and all patients provided their written informed consent.

Blood samples were obtained within 5 minutes after admission to hospital, and the total WBCc was measured in an ethylenediaminetetraacetic acid-anticoagulated whole-blood specimen. To address the confounding effect of other inflammatory factors, C-reactive protein (CRP), d-dimer, and platelet count were also tested simultaneously. We also collected baseline data, including sex, age, duration of pain, and previous medical histories, including hypertension, diabetes mellitus, coronary artery disease, and smoking and drinking status. Other recorded clinical characteristics included baseline vital signs at admission (systolic/diastolic blood pressure, heart rates), imaging examinations, medicine treatment, and surgical intervention including emergency and selective surgery. The rationale and strategy of surgical techniques were determined by surgeons in the department of cardiovascular surgery at our hospital.

All patients were followed up during hospitalization and subsequently via outpatient clinical visits or interim telephone calls until the occurrence of death or loss to follow-up. The short-term mortality was defined as death within 30 days after admission to the emergency department. Two cardiologists who were blinded to the study analyses in FuWai Hospital defined the causes of 30-day death as aortic rupture, perioperative death, dissection-related organ ischaemia, or other causes. Long-term mortality was defined as all-cause death after 30 days during follow-up.

### Statistical Analysis

Continuous variables are presented as the mean ± SD or median and the interquartile range (IQR) according to whether the variable conforms to a normal distribution and were compared using Student *t* test or the Mann–Whitney rank-sum test, as appropriate. Categorical data are presented as the numbers and percentages and were compared using the *χ*^2^ or Fisher exact test. Analysis was stratified according to patients with a WBCc >11.0 × 10^9^ cells/L and ≤11 × 10^9^ cells/L as a cutoff vale for normal, according to previous studies.^13^ Short- and long-term clinical outcomes were determined using the Kaplan-Meier method and compared using the log-rank test. Univariate and multivariate Cox proportional hazards analyses were used to analyze the predictive role of WBCc for short- and long-term mortality with hazard ratios (HRs) and 95% confidence intervals (CIs). Three separate multivariable Cox models were constructed with the WBCc entered as continuous data, using a cutoff value of 11.0 × 10^9^ cells/L, or stratified by tertiles. Other variables selected for testing in the multivariate analysis were those with a *P* < 0.05 in the univariate models. The proportional-hazards assumption was assessed using the log-minus-log-survival function and found to graphically hold. A *P* value <0.05 was considered statistically significant. Data were analyzed using SPSS version 19.0 (SPSS Inc, Chicago, IL).

## RESULTS

A total of 570 patients with CT imaging-confirmed diagnosis of type A AAD were enrolled. All patients had a baseline WBCc available, and the follow-up rates were 99.5% at 30 days and 96.8% at 1 year. The average follow-up period was 1.89 years per subject (IQR, 1.06–3.43 years).

Baseline clinical characteristics of patients stratified by WBCc >11.0 × 10^9^ cells/L or ≤11.0 × 10^9^ cells/L are shown in Table 1. Patients with elevated WBCc had higher heart rate, neutrophil-to-lymphocyte ratio, d-dimer, CRP, serum creatinine levels, but had lower platelet counts, platelet-to-lymphocyte ratio, and less frequently received surgical treatment compared with those with normal WBCc (all *P* < 0.05).

**TABLE 1:**
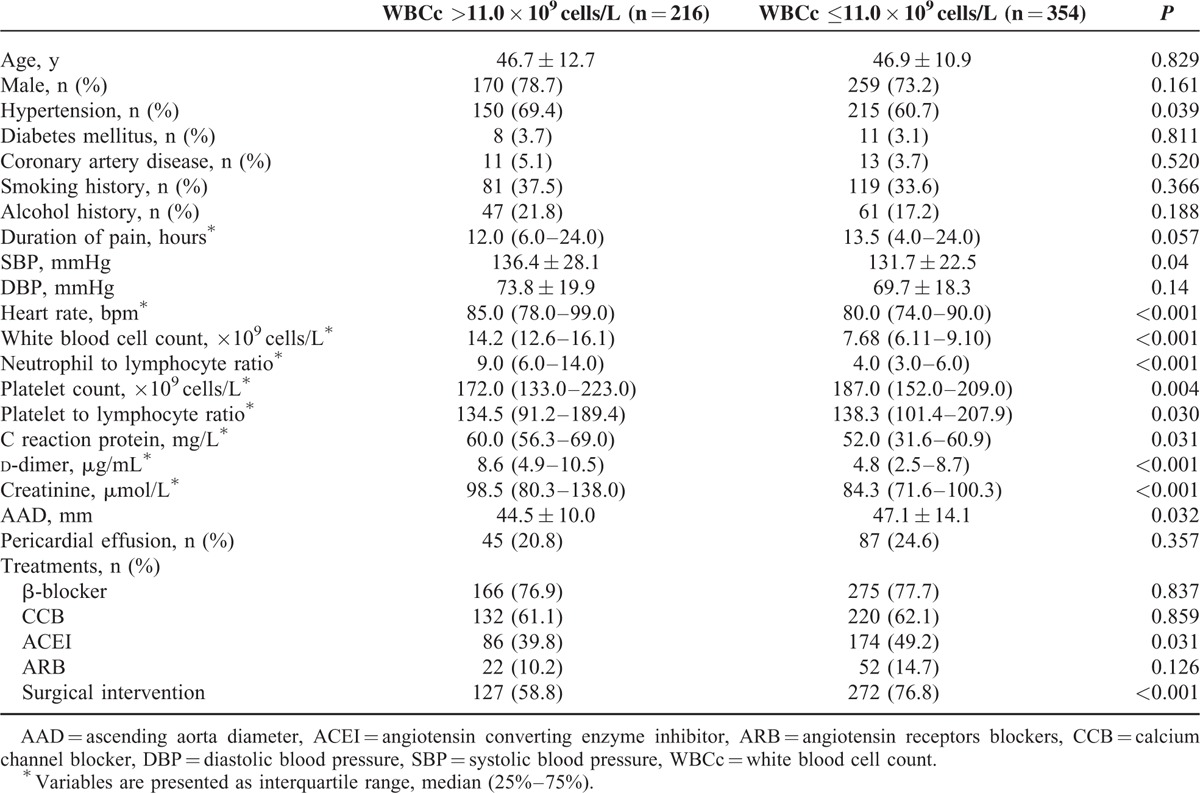
Baseline Characteristics According to Admission White Blood Cell Count

The average hospitalization period was 14 days (IQR, 10–21 days). The overall 30-day mortality was 10.7% (61/570) and 42 patients died of an aortic rupture into the mediastina or pericardial cavity, 9 patients died during the perioperative period, 6 patients died of heart failure, and 4 other patients died from unknown reasons.

**FIGURE 1:**
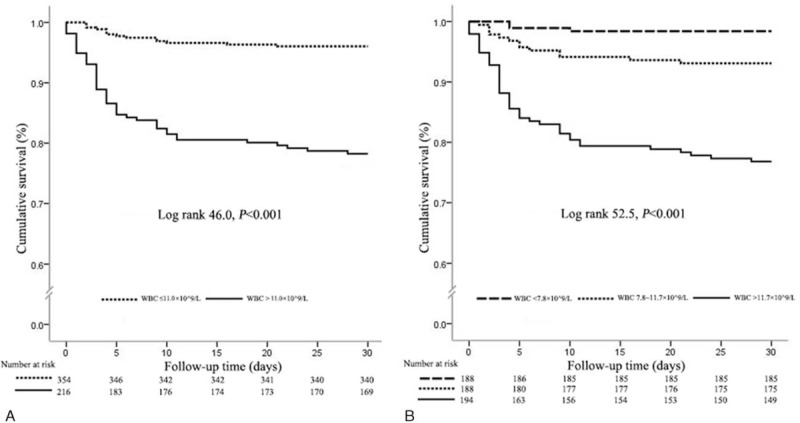
Kaplan-Meier curve for 30-day survival according to the WBCc cutoff value (11.0 × 10^9^ cells/L) and the tertiles of WBCc. (A) Thirty-day survival curves according to the WBCc cutoff value (11.0 × 10^9^ cells/L). (B) Thirty-day survival curves according to the tertiles of WBCc. WBCc = white blood cell count.

As shown in Figure 1A, Kaplan–Meier analysis showed that the cumulative 30-day mortality was significantly higher in patients with elevated admission WBCc compared with those with normal admission WBCc (Log rank *P* < 0.001). When stratified by WBCc tertiles (Figure 1B), the 30-day mortality significantly increased with WBCc increase (Log rank *P* < 0.001).

The results of univariate Cox regression analysis of predictors of 30-day mortality are shown in Table 2. Admission WBCc was associated with 30-day mortality both as a continuous variable (HR = 1.22, 95% CI 1.16–1.27, *P* < 0.001) and as a cutoff value of >11.0 × 10^9^ cells/L (HR = 6.07, 95% CI 3.34–11.1, *P* < 0.001). Other risk factors associated with 30-day all-cause mortality included age, neutrophil–to-lymphocyte ratio, admission d-dimer, CRP, Creatinine, while platelet count, and surgical intervention were protectors for 30-day all-cause mortality.

**TABLE 2:**
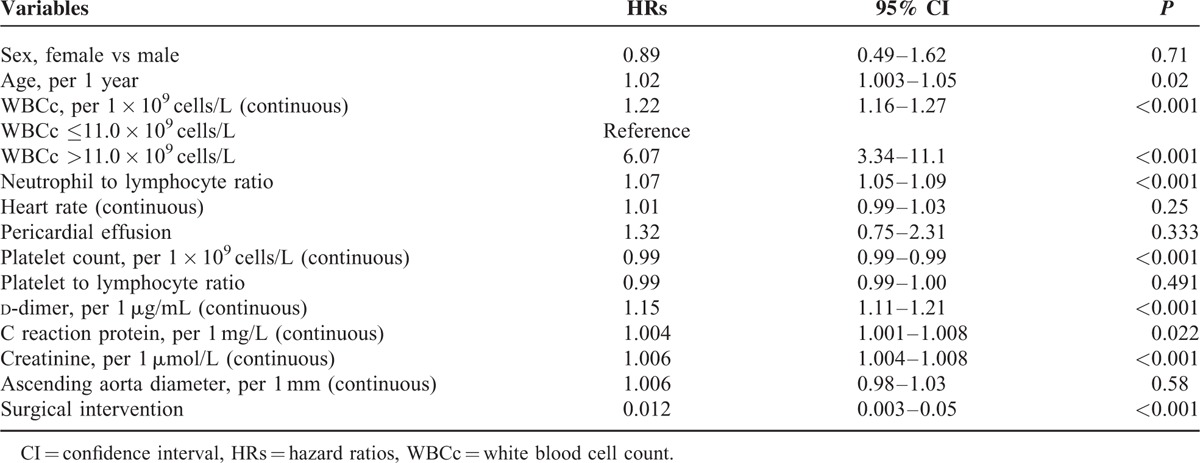
Predictors of 30-Day Death By Univariate Cox Analysis

Multivariable-adjusted HRs for 30-day mortality according to per 1.0 × 10^9^ cells/L increase, the cutoff value of 11.0 × 10^9^ cells/L, and tertiles of WBCc are presented in Table 3. Admission WBCc was an independent predictor of 30-day death when considered as a continuous variable or as a categorical variable using the cutoff value of 11.0 × 10^9^ cells/L or stratified by tertiles (the highest tertile) after adjustment for age, sex, and other inflammatory factors. Surgical intervention was an independent protective factor for 30-day death in 3 Cox models.

**TABLE 3:**
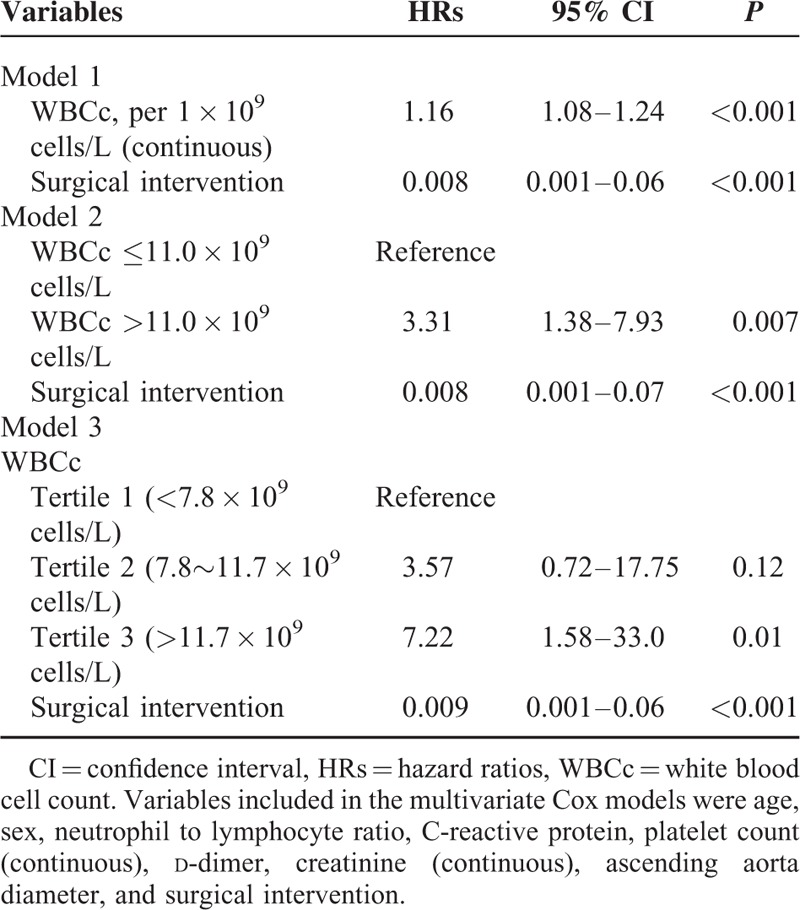
Independent Predictors of 30-Day Death by Multivariable Cox Analysis

**FIGURE 2:**
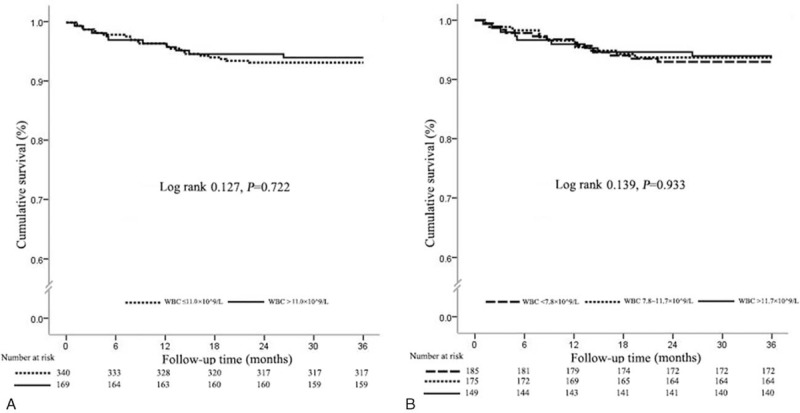
Kaplan-Meier curve for long-term survival according to the WBCc cutoff value (11.0 × 10^9^ cells/L) and the tertiles of WBCc. (A) Long-term survival curves according to the WBCc cutoff value (11.0 × 10^9^ cells/L). (B) Long-term survival curves according to the tertiles of WBCc. WBCc = white blood cell count.

A total of 509 patients were discharged from the hospital. During follow-up, the long-term all-cause mortality was 6.5% (33/509). Kaplan–Meier analysis of the long-term all-cause mortality according to the cutoff value of admission WBCc revealed no significant differences between patients with elevated and normal admission WBCc (Figure 2). Further univariate and multivariable Cox regression analysis confirmed no association between long-term all-cause mortality and admission WBCc (Tables 4 and 5). Surgical intervention was the only factor associated with long-term mortality.

**TABLE 4:**
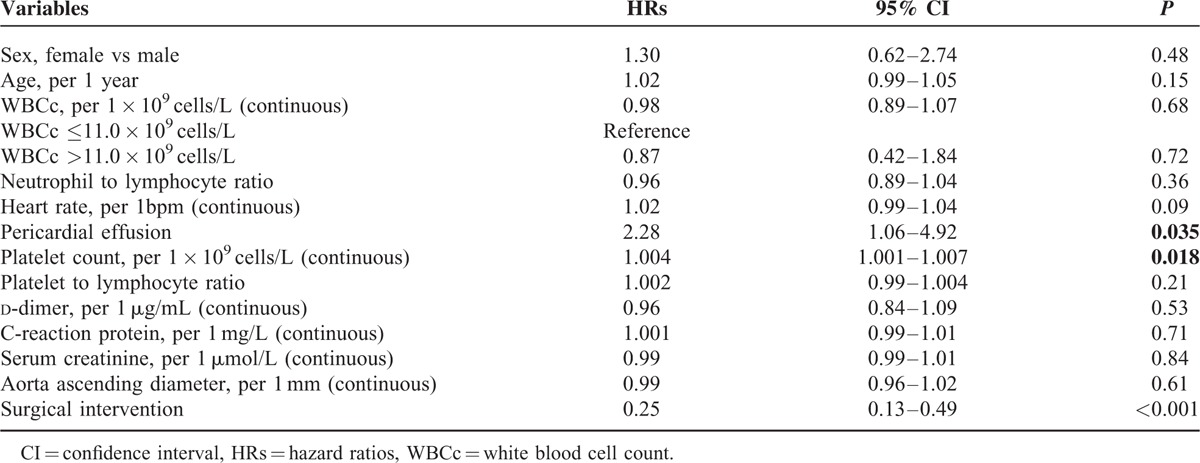
Predictors of Long-Term Death By Univariate Cox Analysis3

**TABLE 5:**
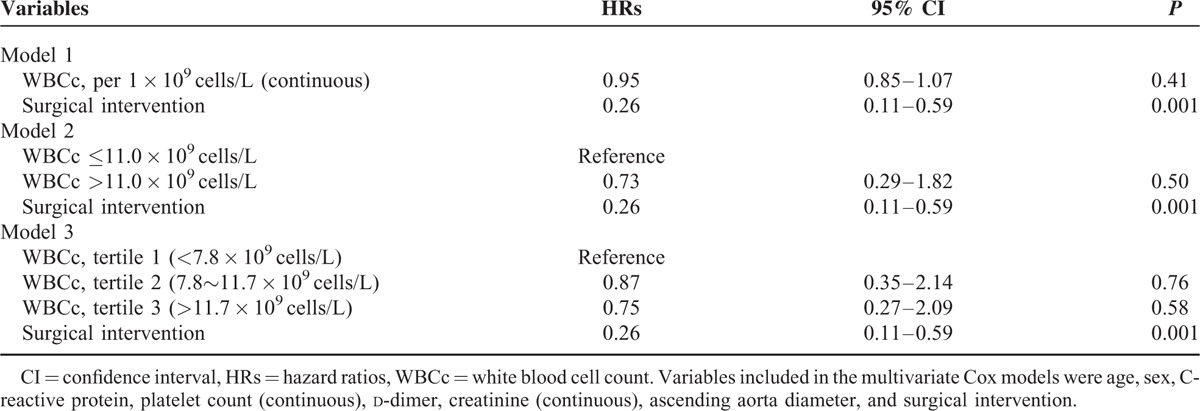
Predictors of Long-Term Death By Multivariate Cox Analysis

## DISCUSSION

The present study, based on a large sample cohort of patients with type A AAD, highlights the relevance of elevated WBCc on admission with 30-day death. The prediction for increased risk of short-term outcomes by elevated admission WBCc in patients with type A AAD even was not affected by surgical intervention, an important protective treatment strategy for type A AAD. The impact of elevated admission WBCc seems to be limited on short-term outcome of AAD, whereas no association was found between the baseline WBCc and long-term all-cause mortality in patients with type A AAD.

Inflammation is involved in medial degradation of the aortic artery, arterial wall remodeling, and contributed to aortic wall weakness.^2,14^ Inflammation biomarkers are associated with acute-phase reactions, complications, and the prognosis of AAD. Elevated d-dimer level has been reported to be associated with increased in-hospital mortality.^6,8,15^ CRP, a nonspecific, but sensitive inflammatory marker, is associated with increased in-hospital death in type A AAD^6^ and its peak level is also reported to be associated with the long-term events in patients with type B AAD.^7^

An elevated WBCc has been observed in patients with AAD in previous studies.^8,11,16,17^ Only 2 studies with small sample size reported discrepant results regarding the association between elevated WBCc and in-hospital mortality.^8,11^ In 36 Chinese patients with type A AAD,^11^ an elevated WBCc (≥12.16 × 10^99^ cells/L) was associated with an increased risk of in-hospital death (relative risk = 1.387, 95% CI 1.027–1.875, *P* = 0.033). The other study reported no association between admission WBCc and in-hospital mortality in patients with AAD (n = 94).^8^ However, patients recruited in that study were western with both type A and type B AAD, and the study was a retrospective design with a relatively small sample size. In contrast, our study prospectively enrolled a relatively larger sample size of Chinese patients with only type A AAD. The differences in genetic background, study design, and sample size may partly interpret the inconsistent results. Moreover, our results also confirmed previous findings that elevated admission WBCc was associated with increased short-term death in Chinese patients with type A AAD.^11^ A novel finding of our study is that the association between short-term mortality and elevated admission WBCc was independent of admission CRP, d-dimer levels, and surgical intervention. As the largest study to date addressing the association of admission WBCc with short- and long-term mortality in patients with type A AAD, the present study suggests the utility of WBCc for the identification of patients with type A AAD at high risk of short-term mortality.

An elevated WBCc on admission might be only a high-risk marker, but not directly be responsible for poor short-term outcomes of patients with type A AAD. High WBCc predicts cardiovascular death in patients with myocardial infarction,^18^ atherosclerotic vascular disease,^19^ and peripheral arterial disease^20^ independent of CRP and other confounders. Because WBC may activate increased inflammation, endothelial damage, procoagulant effects, microvascular damage, result in release of vasculotoxic factors, and subsequently lead to increased risk of death in cardiovascular disease,^21^ previous studies suppose that WBCc may play a pathogenic role in vascular diseases. However, the mechanism of AAD might be different from other atherosclerotic vascular disease. In our cohort, only 4.4% patients had concomitant atherosclerosis. An elevated WBCc during acute phase of AAD may just indicate acute inflammatory processes in the dissected aortic wall, the severe extent of aortic damage, and the presence of comorbidities and complications of AAD. Indeed, AAD patients with elevated WBCc had elevated levels of CRP, d-dimer, and creatinine, increased heart rates, and decreased platelet count in our study, which indicated a severe clinical illness condition. Moreover, recent studies have shown that parts of WBC, including neutrophil, lymphocyte, and the ratio of neutrophil and lymphocyte, have some prognostic value in cardiovascular disease.^22–24^ However, our study showed that the total WBCc, not the ratio of neutrophil and lymphocyte, was independently associated with the short-term outcome in patients with type A AAD, which may suggest the admission WBCc might be a more useful prognostic parameter compared with the ratio of neutrophil and lymphocyte in patients with AAD. In addition, no relationship was found between admission WBCc and long-term mortality. Taken together, these data support that elevated WBCc may be a sensitive inflammatory marker reflecting severe extent of type A AAD, and then predict increased risk of short-term death of type A AAD.

The 30-day and long-term mortality in our study were relatively lower compared to previous reports of Western populations. The International Registry of Acute Aortic Dissection study reported a relatively high in-hospital mortality (32.5%) and the long-term mortality (16.2%).^25^ However, after modernizing and standardizing the operative technique and cerebral protection strategies in the last 2 decades, a significant reduction has been observed in operative mortality from 33.9% to 2.8% and a subsequent improvement in the 5-year survival rates from 55% to 85% for type A AAD.^26^ Operative mortality and major morbidity could also be significantly reduced with a high-volume multidisciplinary thoracic aortic surgery team.^27^ The high case volumes of AAD patients in our center (nearly 400 cases per year) help accumulate experience for timely diagnosis of AAD and appropriate medical and surgical treatment, and make the low in-hospital mortality reasonable.^28,29^ About 70% of patients in our study received timely repair of the ascending aorta, which ensures postdischarge survival rates of patients with type A AAD.^25^ In addition, some high-risk patients may have no opportunity to be transferred to our center from local hospitals. Our cohort may consist of relative low-risk patients. Finally, a different genetic background compared with Western populations may partly contribute to the low mortalities of AAD because of the as low as 2.8% and 3.2% operative and in-hospital mortalities reported in other Asian patients with type A AAD.^30^

The present study has several important strengths. First, the reliability of our results was enhanced by our large sample size and the consideration of both short-term and long-term outcomes of AAD. Second, we also considered and adjusted for the impact of surgical treatment and other potential inflammatory biomarkers, including CRP, d-dimer, and platelet count.

Several limitations need to be mentioned. First, this is a single-center observational study. Our results may not be generalized to patients in other centers because the patient volume and experiences in medical treatment and surgical intervention of type A AAD might be significantly different between our center and other centers. Second, our study only analyzed the WBCc measured within 5 minutes on admission. The analysis of a series of WBCc measurements at different time points may provide more valuable information for evaluating the prognostic role of WBCc in AAD. In addition, detailed operative data, including operative procedures and perfusion techniques, might also significantly affect early and late survival of type A AAD. Thus, additional research is needed to understand the role of admission WBCc, alone or in combination with other circulating inflammatory biomarkers, in the risk stratification of AAD.

In conclusion, elevated WBCc on admission may be valuable for prediction of 30-day mortality in patients with type A AAD. The impact of elevated WBCc on short-term mortality of type A AAD is not affected by surgical intervention. No association was found between admission WBCc and long-term mortalities in patients with AAD. These data suggest the utility of inflammatory markers for the identification of patients with AAD at high-risk of short-term mortality.
